# Analysis of the metabolome of *Anopheles gambiae* mosquito after exposure to *Mycobacterium ulcerans*

**DOI:** 10.1038/srep09242

**Published:** 2015-03-18

**Authors:** J. Charles Hoxmeier, Brice D. Thompson, Corey D. Broeckling, Pamela Small, Brian D. Foy, Jessica Prenni, Karen M. Dobos

**Affiliations:** 1 Department of Microbiology, Immunology, and Pathology, Colorado State University, Fort Collins, CO 80523; 2 Proteomics and Metabolomics Facility, Colorado State University, Fort Collins, CO 80523; 3 Department of Microbiology, University of Tennessee at Knoxville, Knoxville, TN 37996; 4 Department of Biochemistry and Molecular Biology, Colorado State University, Fort Collins, CO 80523

## Abstract

Infection with *Mycobacterium ulcerans* causes Buruli Ulcer, a neglected tropical disease. Mosquito vectors are suspected to participate in the transmission and environmental maintenance of the bacterium. However, mechanisms and consequences of mosquito contamination by *M. ulcerans* are not well understood. We evaluated the metabolome of the *Anopheles gambiae* mosquito to profile the metabolic changes associated with bacterial colonization. Contamination of mosquitoes with live *M. ulcerans* bacilli results in disruptions to lipid metabolic pathways of the mosquito, specifically the utilization of glycerolipid molecules, an affect that was not observed in mosquitoes exposed to dead *M. ulcerans*. These results are consistent with aberrations of lipid metabolism described in other mycobacterial infections, implying global host-pathogen interactions shared across diverse saprophytic and pathogenic mycobacterial species. This study implicates features of the bacterium, such as the putative *M. ulcerans* encoded phospholipase enzyme, which promote virulence, survival, and active adaptation in concert with mosquito development, and provides significant groundwork for enhanced studies of the vector-pathogen interactions using metabolomics profiling. Lastly, metabolic and survival data suggest an interaction which is unlikely to contribute to transmission of *M. ulcerans* by *A. gambiae* and more likely to contribute to persistence of *M. ulcerans* in waters cohabitated by both organisms.

Infection with *Mycobacterium ulcerans* results in a necrotizing ulceration of the subcutaneous tissue (Buruli Ulcer disease) and is a major cause of morbidity in more than 30 countries^1^. West and Central Africa, Australia, and similar tropical localities have reported an increasing incidence of the disease over the past decade^2^,^3^. Exposure to the bacteria is thought to occur from a yet unknown, but persistent, environmental niche. After exposure, and over a variable incubation period, infection can progress from a painless nodule, plaque, or edema to severe ulceration. Serological studies of patients in endemic areas indicate high sero-prevelence rates compared to disease incidence rates, suggesting that exposure to the pathogen without the development of disease is common^4^,^5^. It is likely that *M. ulcerans* persists within a complex food web, through the passage and maintenance by various arthropods and mammals within a particular ecosystem^6^. Recently, other non-human mammals have been discovered to be susceptible to infection by *M. ulcerans*, potentially indicating a diversity of reservoirs used by the bacilli to promote persistence in the environment^7^. However, the mechanism used to bridge the environmental reservoir and susceptible vertebrate populations has remained elusive, despite numerous studies.

Several species of insects have been investigated for their ability to maintain and transmit the pathogen, including species in several genera of mosquitoes (*Culex, Anopheles, and Aedes*) and predatory water bugs in the families *Naucoridae* and *Belostomatidae*^8^,^9^. Epidemiological studies have reported strong associations between *M. ulcerans* and mosquitoes in endemic areas, with Buruli Ulcer patients often recalling mosquito bites after visits to endemic areas^10^. Thus, a close association with insects has been proposed as a potential source of infection^11^. Before vector-borne transmission was suspected by investigators, it was widely believed that the acid-fast bacilli (AFB) could be introduced into a previously existing cut or abrasion and subsequently result in Buruli Ulcer disease^12^. This mode of exposure was deemed unlikely in a study by Williamson (2014), which demonstrated a lack of pathology associated with *M. ulcerans* infection when abraded guinea pig skin was contaminated with a suspension of *M. ulcerans*^13^. This study also suggested that the mechanism of exposure to most likely result in classical Buruli Ulcer disease was via direct injection of the bacteria into the skin, further implicating vector-borne transmission.

It is well known that mycobacteria employ a diverse set of virulence determinants promoting their persistence in the environment and the host^14^, many of which are homologous in *M. ulcerans*^15^. The polyketide toxin mycolactone is the primary virulence factor encoded by *M. ulcerans*, however, the mechanisms employed by the bacilli to colonize a particular environmental or invertebrate niche are not well understood. It is likely that additional virulence factors, not limited to mycolactone, participate in the survival of the bacilli in these varied environments^16^. Indeed, expression of mycolactone is not required for colonization in some invertebrate models^17^.

Complex host-pathogen interactions have been thoroughly researched in other models of vector-borne diseases, describing intricate host-immune and metabolic disruptions leading to survival and subsequent transmission of pathogens by insect vectors^18^. This interaction is influenced by the physiology of both the pathogen and the vector, resulting in intertwined metabolism. An intriguing and valuable research objective investigating the mechanisms of pathogen survival in the host and the dynamics of this interaction have led to innovative strategies for vector and pathogen control^19^.

*Anopheles gambiae*, the primary malaria vector in sub-Saharan Africa, is well-known for its ability to transmit *Plasmodium falciparum*, and species of the *A. gambiae* complex are distributed throughout geographic locations endemic for Buruli Ulcer disease^20^. Use of bed nets also correlates with reduced incidence of Buruli Ulcer disease in African foci^12^. In Australia, sympatry between Anopheles mosquitoes and endemicity of Buruli Ulcer disease has been documented, whereby *M. ulcerans* DNA was detected in wild-caught mosquitoes from endemic regions but not in wild-caught mosquitoes from non-endemic regions^1^,^10^. Taken together, these studies suggest an interaction of *M. ulcerans* and *Anopheles* mosquitoes are occurring at some trophic level.

The objective of this study was to examine the interaction between *M. ulcerans* and the *A. gambiae* mosquito. To evaluate the interaction between these two organisms, *A. gambiae* larvae were allowed to develop in water containing live *M. ulcerans*, dead (γ-irradiated) *M. ulcerans*, or without supplemental bacteria. Upon emergence, the metabolic patterns of adult mosquitoes were analyzed to investigate *M. ulcerans* associated effects on development, and to determine if any of these effects were unique to replicating bacilli. Metabolic profiling using non-targeted ultra-high-performance liquid-chromatography coupled tandem mass spectrometry (UPLC-MS/MS) was used to identify novel metabolic biomarkers of exposure to the pathogen. The use of a non-targeted approach enables holistic detection of metabolites resulting in a metabolic fingerprint associated with a specified treatment or exposure. This approach has been used to evaluate metabolic perturbations in other pathogenic and nonpathogenic disease states, and represents an extremely sensitive and powerful analytical tool^21^. Compared to other analytical approaches used in metabolomics studies (e.g. gas chromatography mass spectrometry and nuclear magnetic resonance) UPLC-MS/MS enables the most versatility when interrogating a sample set containing analytes of diverse molecular characteristics^22^. Identified molecules are mapped to known metabolic pathways to assist in the understanding of biological interactions. An understanding of the specific metabolic affects elicited during mosquito maturation concomitant with exposure to live *M. ulcerans* will provide significant insight into dynamics of this host-pathogen interaction and clues towards the role of *A. gambiae* as a reservoir for and persistence of *M. ulcerans* in endemic areas.

## Methods

### Bacterial strain and culture

*Mycobacterium ulcerans* strain 1615::TN*118* GFP^17^ (*M. ulcerans* 1615-GFP) was propagated at 32°C for a period of 3 weeks on Middlebrook 7H11 (Difco Laboratories, Detroit, MI) plates supplemented with Kirschner Selecta-Tabs (Mast Group, Merseyside, UK) and 10 µg/ml kanamycin (7H9+), then aliquoted into infectivity stocks at a concentration of 10^10^ cells/ml. Contaminating bacteria from mosquito samples were acquired by vortexing the tissues of interest in 7H9+ liquid media, performing a 10-fold serial dilution and plating the dilutions on Middlebrook 7H11 plates with similar antibiotic supplements.

### Mosquito species and maintenance

1st-instar larval *Anopheles gambiae* mosquitoes were acquired from the colony maintained at the Arthropod-borne Infectious Disease Laboratory (AIDL) at Colorado State University. 100 larval mosquitoes were distributed to individual cages containing 250 ml of sterile water and supplemented daily with finely ground fish food. All cages were setup in duplicate for the individual treatment groups and each experiment was repeated in triplicate. Mosquitoes were monitored daily and allowed to develop over a period of 10 days in a controlled environment of 28°C and 70% humidity. Mosquito cages were supplemented with sterile water and raisins, ad libitum, upon emergence of adult insects. Upon termination of the study, adult mosquitoes were aspirated from the cages and briefly knocked down at 4°C. 10 female mosquitoes each were distributed for the generation of biological replicates and their use in subsequent assays.

### Invertebrate infections

100 1st-instar larval mosquitoes were exposed, in duplicate, to live or dead supplemental *M. ulcerans* bacteria, or no supplemental bacteria. 10^2^ cells/ml of live *M.ulcerans* 1615-GFP, or 10^2^ cells/ml of dead, γ-irradiated *M. ulcerans* 1615-GFP were added to the sterile water upon the addition of larval mosquitoes. All groups received approximately 100 mg of fish food daily, in addition to the single initial dose of supplemental bacteria. Contaminating bacteria were detected on mosquito tissues via culture, as described above, and immunofluorescence (IFA). 10 larval and 10 adult female mosquitoes from each treatment group were subjected to IFA screening of internal and external tissues for contamination by *M. ulcerans*. The head, midgut, and salivary glands of adult female mosquitoes were removed and immediately placed in 4% paraformaldehyde solution for 10 minutes for fixation and washed in phosphate buffered saline (PBS). After fixation, the tissues were permeabilized by the addition of 0.1% Triton X-100 for 10 minutes and washed in PBS. To distinguish between green mosquito auto-fluorescence and the GFP expressing bacilli, tissues were first probed using a polyclonal anti-*M. ulcerans* antisera (BEI Resources, Manassas, VA). The anti-*M. ulcerans* antibody was then probed with a goat anti-rabbit Cy5 labeled antibody (Life Technologies). NucBlue DAPI nuclear stain (Life Technologies) was applied for a period of 5 minutes as a counter-stain. Fluorescently labeled tissues were viewed by epifluorescence microscopy (Olympus, Center Valley, PA) equipped with a standard epifluorescent attachment filter set. Larval mosquitoes were removed from the development cages and immediately placed in 4% paraformaldehyde for fixation. Larval mosquitoes were viewed with epifluorescent microscope scope as previously described, without additional fluorescent labeling. Images are reproduced without alteration besides cropping and adjustment of light intensity. The mosquito's survival to adulthood was measured by counting the number of emerged adults and generating a Kaplan-Meier survival curve among the treatment groups. The relative fitness of emerged adults was evaluated by measuring the wing size of emerged mosquitoes as a proxy for body size. Both wings were removed and measured electronically via the publicly available ImageJ program (imagej.nih.gov/ij/) using a line measurement plugin.

### Extraction of small molecules

Five adult female mosquitoes from each treatment group were aspirated from their cage, immersed in methanol, and stored at −80°C to preserve their metabolic profile. Frozen mosquitoes were then placed in a small Eppendorf tube containing 100 µl of ice cold methanol and homogenized with a handheld Eppendorf homogenizer. The suspension was centrifuged for 10 minutes at 10,000 × g to remove large debris. The supernatant was then pushed through a 0.22 µm filter attached to a 1 ml syringe to remove remaining small debris. The filtered methanol extract was used for LC-MS/MS analysis.

### Mass Spectrometry

Acquisition: 1 µl injections of the filtered methanol extract were performed on a Waters Acquity UPLC system. Separation was performed using a Waters Acquity UPLC T3 column (1.8 µM, 1.0 × 100 mm), using a gradient from solvent A (water, 0.1% formic acid) to solvent B (Acetonitrile, 0.1% formic acid). Injections were made in 100% A, which was held for 1 min, a 12 minute linear gradient to 95%B was applied, and held at 95% B for 3 minutes, returned to starting conditions over 0.05 minutes, and allowed to reequilibrate for 3.95 minutes. Flow rate was constant at 200 µL/min for the duration of the run. The column was held at 50°C and samples were held at 5°C. Column eluent was infused into a Waters Xevo G2 Q-Tof MS fitted with an electrospray source. Data was collected in positive ion mode, scanning from 50–1200 at a rate of 0.2 seconds per scan, alternating between MS and MS^E^ mode. Collision energy was set to 6 V for MS mode, and ramped from 15–30 V for MS^E^ mode. Calibration was performed prior to sample analysis via infusion of sodium formate solution, with mass accuracy within 1 ppm. The capillary voltage was held at 2200 V, the source temp at 150°C, and the desolvation temperature at 350°C at a nitrogen desolvation gas flow rate of 800 L/hr.

Processing: For each sample, raw data files were converted to .cdf format, and matrix of molecular features as defined by retention time and mass (m/z) was generated using XCMS^23^ software in R for feature detection and alignment (R Development Core Team, 2014, Vienna, Austria). Raw peak areas were normalized to total ion signal in R, outlier injections were detected based on total signal and PC1 of principle component analysis. Features were grouped based on a novel clustering tool, RAMClust^24^, which groups features into spectra based co-elution and covariance across the full dataset, whereby spectra are used to determine the identity of observed compounds in the experiment. Compounds were annotated based on spectral matching to in-house, NISTv12, Metlin, and Massbank metabolite databases. The peak areas for each feature in a spectrum were condensed via the weighted mean of all features in a spectrum into a single value for each compound. The relative quantity (abundance) of each compound was determined by the calculating the mean area of the chromatographic peak among replicate injections (n = 2). This condensed dataset was subjected to statistical analysis.

### Statistical analysis

The processed data were interrogated using principle component analysis (PCA) for dimensionality reduction and to determine the relative influence of the experimental design on the metabolome. All compounds were analyzed by one way ANOVA (*p* ≤ 0.05 following Bonferonni-Hochburg correction considered significant) to generate a dataset containing only compounds that demonstrated statistically significant abundance among all treatment groups. From this dataset, the abundance of individual compounds was compared between treatments using a two-sided student's t-test (p ≤ 0.05) to generate a dataset containing compounds dually significant by both ANOVA and t-test. These dually significant compounds were then subjected to spectral library searching for identification.

### Library searching and compound identification

MS/MS spectra from significant compounds were compared against in-house small molecule libraries developed and validated by the Proteomics and Metabolomics Core facility (PMF) at Colorado State University, the Metlin Mass Spectral database, and NISTv12 for identification via spectral matching and retention time data (when available). Annotation confidence levels as recommended by the Metabolites Standards Initiative were applied^25^. Briefly, samples were initially compared against the PMF validated chemical reference library. A compound was identified with level I confidence upon matching the mass spectra, retention time, and *m/z* of the chemical reference standard validated with identical instrumental conditions. Compounds annotated with level II confidence are based upon similarity of mass spectra, exact mass, and m/z of the putative compound with the commercialized Metlin database and NISTv12. Level III identification was based on similarity of the mass spectra of putative compounds to known compounds in a chemical class. Unknown compounds, which were still differentiated and quantified using the techniques described, contain unique chromatographic features and are reported as “unknown”. Raw mass spectral data is included online as Supplementary Data S3 (mspLib file).

## Results and Discussion

### Time course of mosquito exposure and survival

1^st^ instar *A. gambiae* larvae were distributed into cages and mosquitoes were allowed to develop over a period of 10 days until they emerged as adults. Following exposure to either live or γ-irradiated *M. ulcerans*, mosquitoes were examined by IFA and culture for *M. ulcerans* colonization and mosquito survival was measured via daily counts of larvae. Upon emergence, the number of mosquitoes which survived to adulthood was counted to evaluate survival over the duration of development. Mosquitoes exposed to live *M. ulcerans* demonstrated bacterial colonization by culture and IFA (Supplementary Figure S1). Those mosquitoes also demonstrated reduced survival and fitness, compared to the control groups, as was evidenced by reduced pupation and adult emergence rates (Supplementary Figure S2) and reduced size (winglength) of those that did emerge into adults. (Supplementary Figure S3)

### Composition and analysis of methanol extracts of mosquito after exposure to *M. ulcerans*

Adult mosquitoes were collected from each treatment group, homogenized in cold methanol, and the lysates subjected to UPLC-MS for semi-quantitative global detection of metabolites. Preservation of the metabolite profile is paramount in any metabolomics study, hence the use of rapid freezing and methanol quenching.

**Figure 1:**
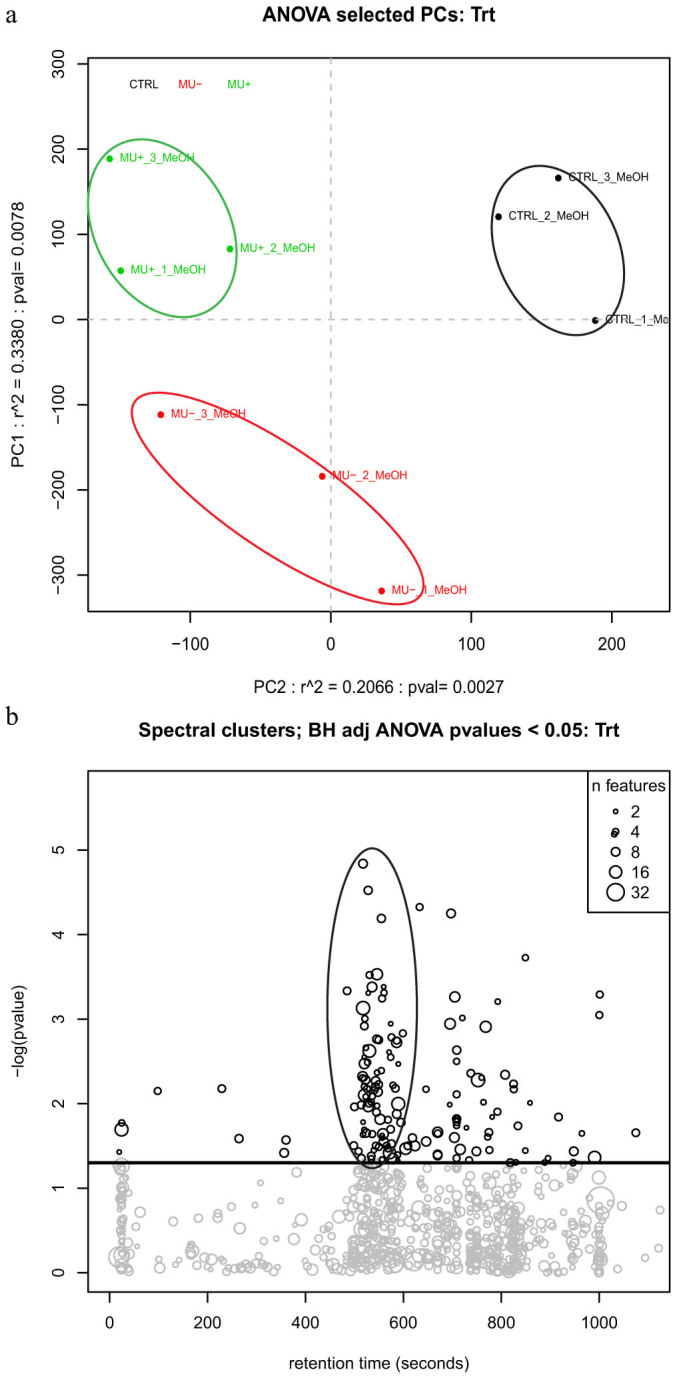
(a) PCA plot of untargeted metabolite analysis demonstrates significant clustering of groups based on treatment. Control group in black (upper right); dead *M. ulcerans* group in red (bottom left); and live *M. ulcerans* group in green (top left); (b) Bubble plot of total metabolites demonstrates spectral clustering of compounds for all treatment groups. Line separating chart represents P<0.05 cutoff for significance. Oval highlighting significant cluster of features as a function of retention time.

XCMS-derived features were clustered via RamclustR^24^ to reduce dataset redundancy and enable spectral-based annotation efforts. Multivariate analysis (PCA) of the clustered metabolite data demonstrates distinct separation of metabolic profiles among treatment groups (Figure 1a). Treatment groups are well separated whereas replicates within each treatment are closely clustered. A bubble plot of p-value as a function of retention time illustrates the large number of metabolites detected in this experiment and highlights clusters of significant features (Figure 1b). 134 compounds were found to have statistically significant differences in abundance among treatment groups (Supplementary Table S4). From this list, 20 were identified with level I confidence^25^ (Table 1). Many compounds were not identifiable with Level I confidence, likely due in part to a lack of coverage of *Anopheles*-specific metabolites in spectral libraries. While metabolomics is quickly becoming a valuable tool for global assessment of exposure-induced metabolic effects, the confident identification of small molecules and the assignment of biological significance represents a bottleneck in the analyses of these studies^26^. The expansion of spectral libraries and the availability of open-source metabolomics data repositories will aid the analysis of future applications of metabolomics. Furthermore, this study adds significant information to the spectral libraries of *Anopheles* mosquitoes.

### Phospholipid pathways affected by exposure to *M. ulcerans*

**Figure 2:**
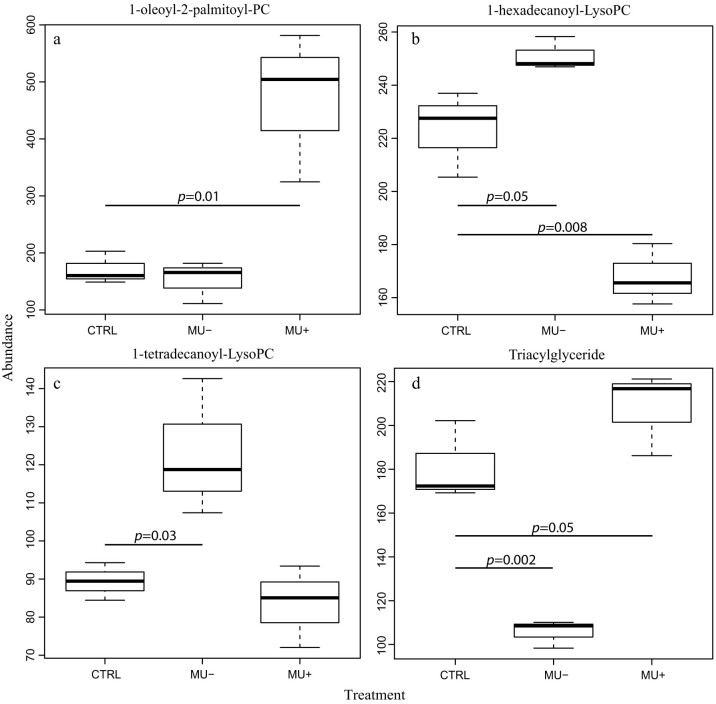
Box and whisker plots of the relative abundance of selected glycerolipid compounds compared by treatment groups. CTRL: control; Mu-: irradiated *M. ulcerans*; Mu+: live *M. ulcerans*.

Analysis of the metabolites found to significantly differ in abundance among treatment groups was performed to correlate the disruption of metabolic pathways with the reduced survival and fitness described in the adult mosquitoes after exposure to the pathogen. Of the identified compounds with significantly different accumulation among treatments, the vast majority belong to the lipid metabolism functional group. This diverse class of molecules is involved in signaling and as mediators in numerous cellular and immune processes. In particular, phospholipids represent the main components of biological membranes^27^. In contaminated *A. gambiae*, diacyl glycerophosphocholine (PC) molecules were found to be increased in abundance in live *M. ulcerans* exposure groups (Table 1). Specifically, the accumulation of 1-oleoyl-2-palmitoyl-PC in the live *M. ulcerans* groups compared to both the control and the γ-irradiated *M. ulcerans* groups suggests a mechanism of live-pathogen induced disruption of the utilization of phosphatidylcholine compounds (Figure 2a).

Accumulation of lyso-phosphatidylcholine compounds (LysoPC) (monoacylated glycerophosphocholine) occurs primarily in mosquitoes exposed to dead *M. ulcerans* compared to live *M. ulcerans* treatment groups. This finding, along with that of higher PC abundance in mosquitoes exposed to live bacilli, suggests hydrolysis of the parent PC is interrupted, resulting in reduced lyso-PC in colonized mosquitoes. Alternatively, this finding may suggest that mosquitoes exposed to live pathogen have increased biochemical activity associated with metabolism of this compound or alternatively exhibit disrupted synthesis pathways compared to control groups^28^ (Figure 2b–c).

**Figure 3:**
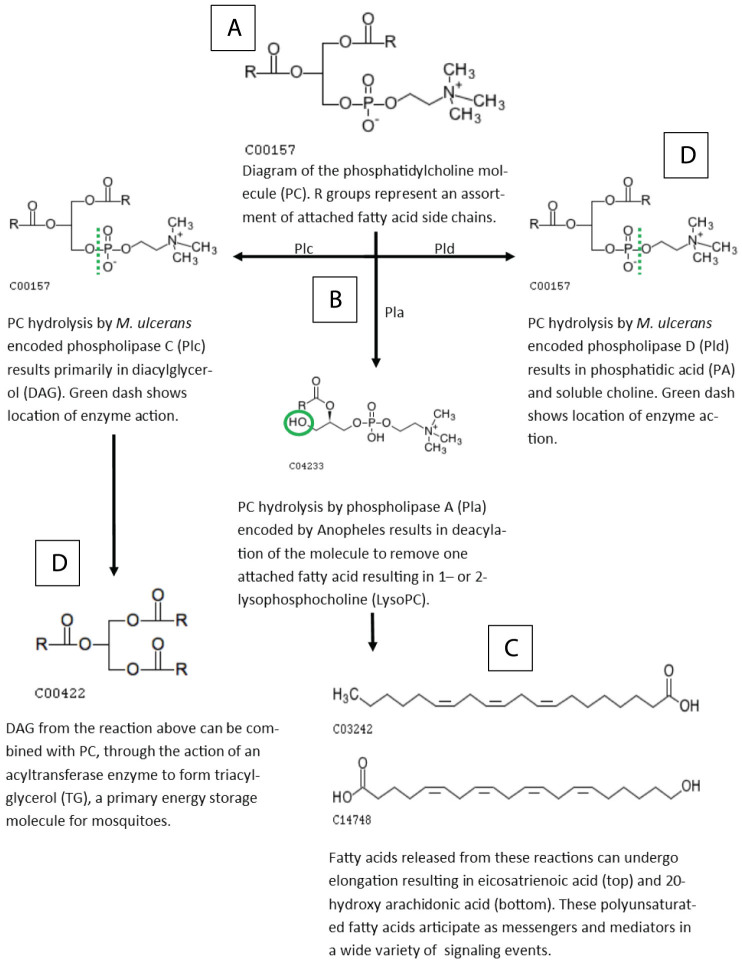
Phospholipids (PL) are a major constituent of biological membranes. PL are characterized by a glycerol backbone attached to a phosphodiester group and a polar head group. Phosphatidylcholine (PC) is a functional class of PL molecule characterized by a choline head group. The fatty acid composition of PC can vary, but is generally composed of one saturated fatty acid and one unsaturated fatty acid, attached in the R position (A). Phospholipase hydrolysis of the PC molecule results in a plethora of subunits involved in many downstream signaling and metabolic processes (B). As PC molecules are cleaved by phospholipase A/B/C/D (Pla/b/c/d) (hashed lines), fatty acids are released (C), in addition to other cleavage products such as diacylglycerol (DAG), phosphatidic acid (PA), and choline (D). Fatty acids liberated from PC, DAG, TG, LysoPC can be elongated and/or modified to form ubiquitous signaling molecules. In this study, eicosatrienoic acid and 20-hydroxy eicosatetraenoic acid (hydroxy arachidonic acid) were found to be in lower abundance in groups exposed to M. ulcerans than controls. Molecular diagrams from Kyoto Encyclopedia of Genes and Genomes (www.Kegg.jp/kegg/) [50, 51].

Glycerolipid metabolism in Anopheles mosquitoes is a critical component of metabolism utilized for the generation of lipid energy sources, components of cell membranes, and signaling pathway modulators^29^. Mosquitoes rely on a lipid carrier protein, lipophorin, as a reusable shuttle for the transportation of lipid molecules from sites of storage or synthesis to sites of utilization as an energy source or as precursors to triacylglycerol and phospholipid synthesis. In contrast with other eukaryotic organisms which store lipids as mixtures of mono-, di-, and triglycerides; triacylglycerides (TG) are the major component of mosquito lipid storage and may be cleaved to release fatty acids as an energy source^29^ (Figure 3d). In this study, TG were found to significantly increase in abundance in mosquitoes exposed to live *M. ulcerans;* whereas mosquitoes exposed to dead *M. ulcerans* had lower abundance of TG than control mosquitoes (Figure 2d).

Disruption of host-lipid metabolism is a well-characterized feature of many mycobacterial infections, as well as in an increasing number of intra- and extracellular pathogens including *Clostridium perfringens, Corynebacterium pseudotuberculosis, Pseudomonas aeruginosa, Staphylococcus aureus,* *Listeria monocytogenes, M. leprae*, and *M. avium*^30^. Additionally, histopathological analysis of mouse footpad tissues revealed the presence of foamy macrophages during late *M. ulcerans* infection (unpublished observations), a feature indicative of aberrant host metabolism^31^. During active and chronic infection with *M. tuberculosis*, the accumulation of TG-rich lipid bodies in foamy macrophages is used as an energy source for the intracellular pathogen^32^. The development of *P. falciparum* in infected mosquitoes is also associated with disruption of host lipid metabolic pathways, thus our findings appear consistent with mechanisms employed by other pathogens for survival in the mosquito^33^. Specifically, the observed increase in abundance of TG in mosquitoes exposed to live *M. ulcerans*, but not γ-irradiated *M. ulcerans* (Figure 2d), is consistent with these trends and has not yet been described in the mosquito system.

### Fatty acid mediators and signaling

**Figure 4:**
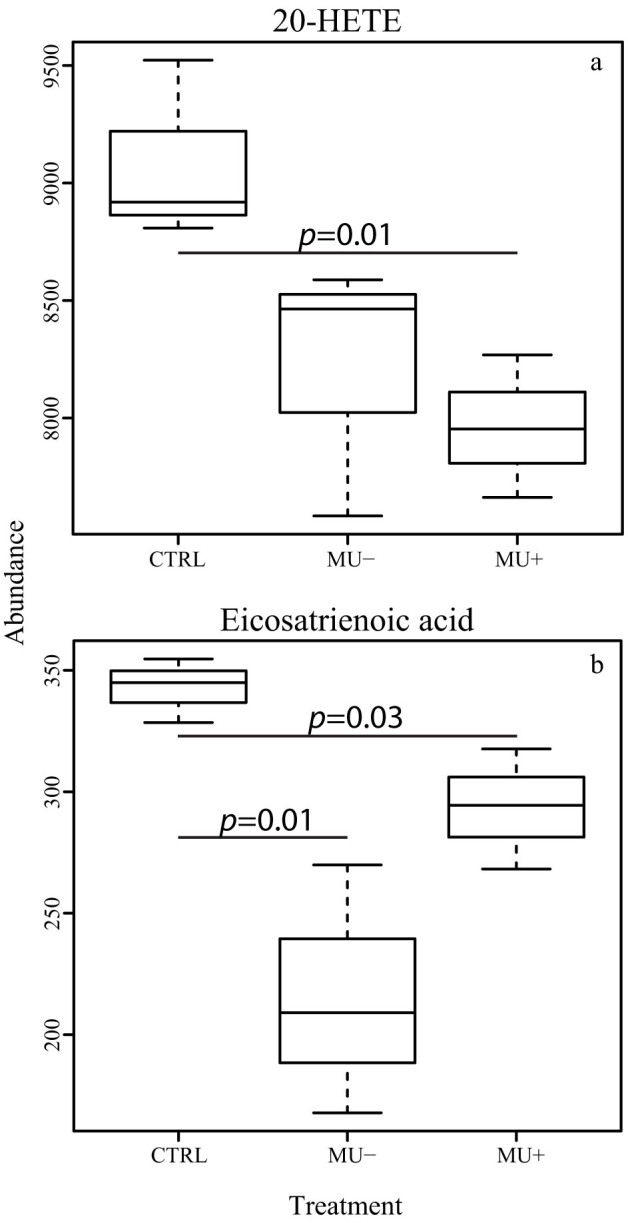
Abundance of eicosanoid compounds in mosquitoes exposed to live (Mu+) and dead (Mu-) *M. ulcerans* bacteria compared to control mosquitoes. Eicosanoids are mediators of a wide variety of immune signaling processes, and the down regulation of these compounds is suspected to increase the survival of contaminating pathogens.

Mosquitoes treated with M. ulcerans were found to have significantly lower abundance of the eicosanoids 8,11,14-Eicosatrienoic acid and 20-Hydroxy-(5Z,8Z,11Z,14Z)- eicosatetraenoic acid (20-HETE) (Figure 4a–b). Eicosanoids are oxygenated metabolites of polyunsaturated fatty acids and are potent signaling molecules involved in inflammation, immunity and the nervous system of vertebrates, invertebrates, and many eukaryotic microbes^34^. For example, the compound 20-HETE, a metabolite of arachidonic acid, is involved in the detoxification response via the cytochrome P450 system, a pathway which contributes to metabolic resistance to insecticides in *Anopheles* mosquitoes^35^ Interestingly, suppression of eicosanoid synthesis within the host is a major mechanism utilized by entomopathogenic bacteria during infection and during protozoan development in *Anopheles* mosquitoes^36^, suggesting that modulation of eicosanoid signaling by pathogenic contaminants of invertebrate systems facilitates survival of these pathogens.

### Secondary metabolites

In addition to the various perturbations of lipid metabolic pathways discovered in this study, two other compounds deserve mentioning. However, the analysis of their role in our model of infection is undertaken with restraint due to the lack of available information regarding the dynamics of these molecules in the *Anopheles* mosquito. Insects use a variety of pheromones during reproduction and host-seeking behavior. The codling moth (*Cydia pomonella*) produces dodecadienol as a precursor in the synthesis of a sex hormone^37^. The presence of this compound has not yet been described in mosquito systems; none the less 8-10-dodecadienol was identified in this study, and a significant reduction of the metabolite was found in mosquitoes exposed to both live and dead *M. ulcerans* compared to control groups in our model. This may suggest a novel activity of this compound in mosquitoes potentially affected by bacterial contamination (Table 1).

**Figure 5:**
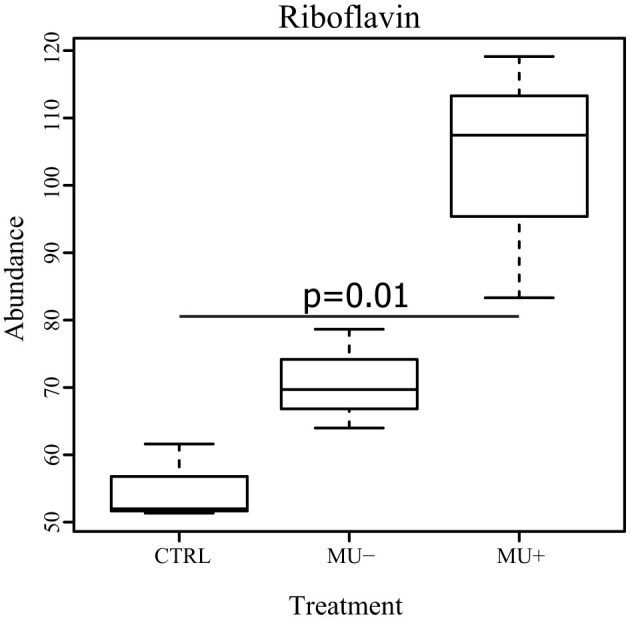
Box and whisker plot showing the abundance of riboflavin in treatment groups. Mosquitoes exposed to live *M. ulcerans* (Mu+) show a significant accumulation of riboflavin compared to the other groups. CTRL: control, and Mu-: dead *M. ulcerans*.

Riboflavin was also identified and found to be significantly increased in mosquitoes exposed to live *M. ulcerans* compared to both control mosquitoes and mosquitoes exposed to dead *M. ulcerans* (Figure 5b). Riboflavin is an essential B vitamin in nearly all pro- and eukaryotic organisms, serving as a precursor for the synthesis of flavin coenzymes and flavin adenine dinucleotide which are essential cofactors for a wide variety of metabolic enzymes and electron transport^38^. In addition to metabolic processes, riboflavin is also involved in areas of yellow pigmentation in the mosquito, such as within the eyes and malpighian tubules^39^. The accumulation of riboflavin in mosquitoes exposed to live *M. ulcerans*, and the reduced accumulation in mosquitoes exposed dead *M. ulcerans* is surprising (Figure 5). *Mycobacterium smegmatis*, and the closely related *Corynebacterium diptheriae*, are considered to be overproducers of riboflavin^38^. Concomitantly, the proteins involved in riboflavin synthesis and processing have been documented in the *M. ulcerans* proteome (NCBI accession: WP_011740179 (riboflavin kinase) and WP_011740778 (riboflavin biosynthesis protein)). The dynamics of riboflavin metabolism are not specifically defined in *M. ulcerans*, however a flavin analog (F420-dependent reductase) is synthesized using a riboflavin precursor and is involved in the degradation of aflatoxins^40^. Evidence from *in vitro* studies suggests that an overproduction or supplementation of riboflavin may be involved in slowing the development of *P. falciparum*^41^, however the role of this compound *in vivo* is uncertain. Thus, the accumulation of riboflavin in our model may be due to an overproduction of the molecule by contaminating bacilli, or a response mechanism by the mosquito due to *M. ulcerans* colonization (Figure 5).

## Conclusion

The compounds identified in our model and the putative assignment of biological significance thereof may represent the cumulative effects of mechanisms employed by *M. ulcerans* to parasitize or adapt to environments cohabited by *A. gambiae* mosquitoes. Many of the detected compounds suggest that disrupted metabolism in the mosquito with exposure to *M. ulcerans* are novel. Consequently, the analyses of the various metabolic processes affected by these compounds are based on analogous mechanisms from similar phenomena observed among other host-pathogen interactions. Furthermore, analogous glycerolipid and phospholipid pathways have been documented to be disrupted by *M. tuberculosis*^42^,^43^ and *M. leprae*^44^ during infection, suggesting global metabolic adaptations to mycobacterial infections across many hosts. From a large number of compounds, few are able to be identified with a high level of confidence, due to limited availability of validated standards and limited understanding of selected metabolic pathways in mosquitoes. For example, an ACTH-like peptide (C449; Supplementary table S2) was confidently identified based on molecular and spectral characteristics, and was found in significantly greater abundance in live *M. ulcerans* exposed groups. However, pathways involved in peptide hormone metabolism in insects are not well understood, thus limiting the discussion of the biological significance of this molecule after exposure to the pathogen. Future studies, aimed at an understanding of which of these adaptive processes are helpful to host survival versus which contribute to pathogen persistence will further our understanding of reservoir maintenance of *M. ulcerans* in endemic areas, and may contribute to the development of novel transmission-blocking interventions applicable to other mosquito-borne diseases.

## Author Contributions

J.C.H., C.D.B., B.D.F. and K.M.D. conceived the experiments, J.C.H., B.D.T. and C.D.B. performed the experiments, J.C.H. and K.M.D. prepared and edited the manuscript text. J.C.H. and C.D.B. prepared the figures. All authors reviewed the manuscript.

## Supplementary Material

Supplementary Information Supplementary Information

## Figures and Tables

**Table 1:** Identification and Annotation of Significant Metabolic Features

ID^a^	Compound Annotation^b^	KEGG ID^c^	Retention time^d^	m/z^e^	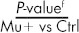	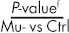	ID confidence^g^	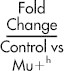	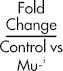
C724	1-oleoyl-2-palmitoyl-sn-glycero-3-PC 18:1/16:0	C04317	759.8	760.597	0.018	0.5	I	2.754	0.896
C295	Riboflavin	C00255	229.1	377.146	0.012	0.043	I	1.879	1.287
C737	GPEtn(18:3(6Z,9Z,12Z)/18:3(6Z,9Z,12Z))	C04475	829.8	737.539	0.055	0.038	I	1.726	1.776
C67	1-oleoyl-2-hydroxy-sn-glycero-3-PC 18:1	C04317	546.1	544.340	0.015	0.018	I	1.359	0.681
C32	GPCho(16:0/16:1(7Z))	C00157	530.5	494.324	0.101	0.005	I	1.241	1.818
C35	1-palmitoyl-2-hydroxy-sn-glycero-3-PC 16:0	C04317	571.6	496.340	0.013	0.091	I	1.237	1.192
C29	1-oleoyl-2-hydroxy-sn-glycero-3-PC 18:1	C04317	589.4	522.356	0.131	0.006	I	1.150	1.332
C512	TG 61:5; [M+Na]+; TG(19:0/20:5/22:0)	C00422	1001.0	703.575	0.050	0.002	I	1.148	0.583
C440	1-oleoyl-2-hydroxy-sn-glycero-3-phosphoethanolamine	C01233	586.4	480.309	0.070	0.004	I	1.117	1.267
C581	Phosphocholine headgroup	C00114	526.9	564.307	0.410	0.010	I	1.099	1.759
C80	1-oleoyl-2-hydroxy-sn-glycero-3-PC 18:1	C04317	578.6	522.356	0.055	0.028	I	1.074	1.118
C810	1-heptadecanoyl-2-hydroxy-sn-glycero-3-PC 17:0	C04317	556.7	473.272	0.971	0.031	I	1.002	1.208
C96	8,11,14-Eicosatrienoic acid	C03242	705.1	305.248	0.639	<0.001	I	0.974	0.684
C401	Adenosine	C00212	98.1	141.959	0.708	0.007	I	0.966	0.642
C800	1-tetradecanoyl-sn-glycero-3-PC 14:0	C04317	517.6	253.603	0.438	0.035	I	0.934	1.375
C179	20-Hydroxy-(5Z,8Z,11Z,14Z)-eicosatetraenoic acid	C14748	559.8	303.232	0.016	0.087	I	0.877	0.904
C787	8,11,14-Eicosatrienoic acid	C03242	694.6	305.248	0.038	0.014	I	0.856	0.629
C808	1-hexadecanoyl-sn-glycero-3-PC 16:0	C04317	559.7	478.327	0.008	0.050	I	0.752	1.125
C766	trans-8, trans-10-Dodecadien-1-ol	C02679	1031.0	141.959	<0.001	<0.001	I	0.467	0.579
C349	trans-8, trans-10-Dodecadien-1-ol	C02679	1074.8	141.959	0.028	0.030	I	0.369	0.398

Key: a) Compound identifier. b) Identification of compound based on match of mass spectra, m/z ratio, and/or retention time to available databases. c) Compound identifier from Kyoto Encyclopedia of Genes and Genomes (www.genome.jp/kegg/) d) Retention time (seconds) generated via liquid chromatography. e) Mass to charge ratio of the base peak from the mass spectra. f) T-test p-value of pairwise comparison between specified treatment and control group. g) Confidence level of identification based on matching of chromatographic and mass spectral characteristics for each compound to validated compound library or publicly available databases (from reference #27). h/i) Fold change in abundance of compound compared between control and treatment groups. Abbreviations: PC: phosphocholine; GPCho: glycerophosphocholine; GPEtn: glycerophosphoethanolamine; GPSer: glycerophosphoserine; ACTH: adrenocorticotropic hormone; X:N represents number of carbons to double bonds in a given compound.

## References

[b1] Johnson P. D. *et al.* Mycobacterium ulcerans in mosquitoes captured during outbreak of Buruli ulcer, southeastern Australia. Emerg Infect Dis 13, 1653–1660 (2007). 18217547 10.3201/eid1311.061369 PMC3375796

[b2] Bayonne Manou L. S. *et al.* [Mycobacterium ulcerans disease (Buruli ulcer) in Gabon: 2005-2011]. Medecine et sante tropicales 23, 450–457, 10.1684/mst.2013.0259 (2013). 24413612

[b3] Morris A. *et al.* First detection of Mycobacterium ulcerans DNA in environmental samples from South America. PLoS Negl Trop Dis 8, e2660, 10.1371/journal.pntd.0002660 (2014). 24498449 PMC3907311

[b4] Pidot S. J. *et al.* Serological evaluation of Mycobacterium ulcerans antigens identified by comparative genomics. PLoS Negl Trop Dis 4, e872, 10.1371/journal.pntd.0000872 (2010). 21072233 PMC2970529

[b5] Dobos K. M., Spotts E. A., Marston B. J., Horsburgh C. R. Jr & King C. H. Serologic response to culture filtrate antigens of Mycobacterium ulcerans during Buruli ulcer disease. Emerg Infect Dis 6, 158–164, 10.3201/eid0602.000208 (2000). 10756149 PMC2640848

[b6] Fyfe J. A. *et al.* A major role for mammals in the ecology of Mycobacterium ulcerans. PLoS Negl Trop Dis 4, e791, 10.1371/journal.pntd.0000791 (2010). 20706592 PMC2919402

[b7] O'Brien C. R. *et al.* Clinical, microbiological and pathological findings of Mycobacterium ulcerans infection in three Australian Possum species. PLoS Negl Trop Dis 8, e2666, 10.1371/journal.pntd.0002666 (2014). 24498451 PMC3907337

[b8] Gamboa M., Kimbirauskas R. K., Merritt R. W. & Monaghan M. T. A molecular approach to identifying the natural prey of the African creeping water bug Naucoris, a potential reservoir of Mycobacterium ulcerans. Journal of insect science 12, 2, 10.1673/031.012.0201 (2012). 22934669 PMC3465933

[b9] Wallace J. R. *et al.* Interaction of Mycobacterium ulcerans with mosquito species: implications for transmission and trophic relationships. Appl Environ Microbiol 76, 6215–6222, 10.1128/AEM.00340-10 (2010). 20675453 PMC2937476

[b10] Lavender C. J. *et al.* Risk of Buruli ulcer and detection of Mycobacterium ulcerans in mosquitoes in southeastern Australia. PLoS Negl Trop Dis 5, e1305, 10.1371/journal.pntd.0001305 (2011). 21949891 PMC3176747

[b11] Rodhain F. [Buruli ulcer: hypothetical modes of transmission of Mycobacterium ulcerans]. Bull Acad Natl Med 196, 685–690; discussion 690–681 (2012). 23472356

[b12] Landier J. *et al.* Adequate wound care and use of bed nets as protective factors against Buruli Ulcer: results from a case control study in Cameroon. PLoS Negl Trop Dis 5, e1392, 10.1371/journal.pntd.0001392 (2011). 22087346 PMC3210760

[b13] Williamson H. R. *et al.* Mycobacterium ulcerans Fails to Infect through Skin Abrasions in a Guinea Pig Infection Model: Implications for Transmission. PLoS Negl Trop Dis 8, e2770, 10.1371/journal.pntd.0002770 (2014). 24722416 PMC3983084

[b14] Gordon S. & Andrew P. W. Mycobacterial virulence factors. Society for Applied Bacteriology symposium series 25, 10S–22S (1996). 8972115

[b15] Roltgen K., Stinear T. P. & Pluschke G. The genome, evolution and diversity of Mycobacterium ulcerans. Infect Genet Evol 12, 522–529, 10.1016/j.meegid.2012.01.018 (2012). 22306192

[b16] Dobos K. M., Small P. L., Deslauriers M., Quinn F. D. & King C. H. Mycobacterium ulcerans cytotoxicity in an adipose cell model. Infection and immunity 69, 7182–7186, 10.1128/IAI.69.11.7182-7186.2001 (2001). 11598099 PMC100123

[b17] Mosi L., Williamson H., Wallace J. R., Merritt R. W. & Small P. L. Persistent association of Mycobacterium ulcerans with West African predaceous insects of the family belostomatidae. Appl Environ Microbiol 74, 7036–7042, 10.1128/AEM.01234-08 (2008). 18836026 PMC2583505

[b18] Sreenivasamurthy S. K. *et al.* A compendium of molecules involved in vector-pathogen interactions pertaining to malaria. Malaria journal 12, 216, 10.1186/1475-2875-12-216 (2013). 23802619 PMC3734095

[b19] Silva M. T., Portaels F. & Pedrosa J. Aquatic insects and Mycobacterium ulcerans: an association relevant to Buruli ulcer control? PLoS medicine 4, e63, 10.1371/journal.pmed.0040063 (2007). 17326706 PMC1805625

[b20] Sinka M. E. *et al.* The dominant Anopheles vectors of human malaria in Africa, Europe and the Middle East: occurrence data, distribution maps and bionomic precis. Parasites & vectors 3, 117, 10.1186/1756-3305-3-117 (2010). 21129198 PMC3016360

[b21] Broeckling C. D., Heuberger A. L. & Prenni J. E. Large scale non-targeted metabolomic profiling of serum by ultra performance liquid chromatography-mass spectrometry (UPLC-MS). J Vis Exp, e50242, 10.3791/50242 (2013). 23524330 PMC3639512

[b22] Gika H. G., Theodoridis G. A., Plumb R. S. & Wilson I. D. Current practice of liquid chromatography-mass spectrometry in metabolomics and metabonomics. Journal of pharmaceutical and biomedical analysis 87, 12–25, 10.1016/j.jpba.2013.06.032 (2014). 23916607

[b23] Tautenhahn R., Bottcher C. & Neumann S. Highly sensitive feature detection for high resolution LC/MS. BMC Bioinformatics 9, 504, 10.1186/1471-2105-9-504 (2008). 19040729 PMC2639432

[b24] Broeckling C. D., Afsar F. A., Neumann S., Ben-Hur A. & Prenni J. E. RAMClust: A Novel Feature Clustering Method Enables Spectral-Matching-Based Annotation for Metabolomics Data. Analytical chemistry 86, 6812–6817, 10.1021/ac501530d (2014). 24927477

[b25] Sumner L. W. *et al.* Proposed minimum reporting standards for chemical analysis Chemical Analysis Working Group (CAWG) Metabolomics Standards Initiative (MSI). Metabolomics : Official journal of the Metabolomic Society 3, 211–221, 10.1007/s11306-007-0082-2 (2007). 24039616 PMC3772505

[b26] Hollywood K., Brison D. R. & Goodacre R. Metabolomics: current technologies and future trends. Proteomics 6, 4716–4723, 10.1002/pmic.200600106 (2006). 16888765

[b27] Piotto S. *et al.* The effect of hydroxylated fatty acid-containing phospholipids in the remodeling of lipid membranes. Biochimica et biophysica acta 1838, 1509–1517, 10.1016/j.bbamem.2014.01.014 (2014). 24463068

[b28] D'Arrigo P. & Servi S. Synthesis of lysophospholipids. Molecules 15, 1354–1377, 10.3390/molecules15031354 (2010). 20335986 PMC6257299

[b29] Atella G. C. & Shahabuddin M. Differential partitioning of maternal fatty acid and phospholipid in neonate mosquito larvae. The Journal of experimental biology 205, 3623–3630 (2002). 12409488 10.1242/jeb.205.23.3623

[b30] Raynaud C. *et al.* Phospholipases C are involved in the virulence of Mycobacterium tuberculosis. Mol Microbiol 45, 203–217 (2002). 12100560 10.1046/j.1365-2958.2002.03009.x

[b31] Singh V. *et al.* Mycobacterium tuberculosis-driven targeted recalibration of macrophage lipid homeostasis promotes the foamy phenotype. Cell host & microbe 12, 669–681, 10.1016/j.chom.2012.09.012 (2012). 23159056

[b32] Caire-Brandli I. *et al.* Reversible lipid accumulation and associated division arrest of Mycobacterium avium in lipoprotein-induced foamy macrophages may resemble key events during latency and reactivation of tuberculosis. Infect Immun 82, 476–490, 10.1128/IAI.01196-13 (2014). 24478064 PMC3911402

[b33] Vlachou D., Schlegelmilch T., Christophides G. K. & Kafatos F. C. Functional genomic analysis of midgut epithelial responses in Anopheles during Plasmodium invasion. Current biology : CB 15, 1185–1195, 10.1016/j.cub.2005.06.044 (2005). 16005290

[b34] Stanley D. Prostaglandins and other eicosanoids in insects: biological significance. Annu Rev Entomol 51, 25–44, 10.1146/annurev.ento.51.110104.151021 (2006). 16332202

[b35] Stevenson B. J. *et al.* Cytochrome P450 6M2 from the malaria vector Anopheles gambiae metabolizes pyrethroids: Sequential metabolism of deltamethrin revealed. Insect biochemistry and molecular biology 41, 492–502, 10.1016/j.ibmb.2011.02.003 (2011). 21324359

[b36] Hwang J., Park Y., Kim Y., Hwang J. & Lee D. An entomopathogenic bacterium, Xenorhabdus nematophila, suppresses expression of antimicrobial peptides controlled by Toll and Imd pathways by blocking eicosanoid biosynthesis. Archives of insect biochemistry and physiology 83, 151–169, 10.1002/arch.21103 (2013). 23740621

[b37] El-Sayed A., Liblikas I. & Unelius R. Flight and molecular modeling study on the response of codling moth, Cydia pomonella (Lepidoptera: Tortricidae) to (E,E)-8,10-dodecadien-1-ol and its geometrical isomers. Zeitschrift fur Naturforschung. C, Journal of biosciences 55, 1011–1017 (2000). 10.1515/znc-2000-11-1226 11204178

[b38] Abbas C. A. & Sibirny A. A. Genetic control of biosynthesis and transport of riboflavin and flavin nucleotides and construction of robust biotechnological producers. Microbiology and molecular biology reviews : MMBR 75, 321–360, 10.1128/MMBR.00030-10 (2011). 21646432 PMC3122625

[b39] Nickla H. Interaction between pteridine synthesis and riboflavin accumulation in Drosophila melanogaster. Canadian journal of genetics and cytology. Journal canadien de genetique et de cytologie 14, 105–111 (1972). 4624271 10.1139/g72-013

[b40] Lapalikar G. V. *et al.* F420H2-dependent degradation of aflatoxin and other furanocoumarins is widespread throughout the actinomycetales. PLoS One 7, e30114, 10.1371/journal.pone.0030114 (2012). 22383957 PMC3288000

[b41] Akompong T., Eksi S., Williamson K. & Haldar K. Gametocytocidal activity and synergistic interactions of riboflavin with standard antimalarial drugs against growth of Plasmodium falciparum in vitro. Antimicrob Agents Chemother 44, 3107–3111 (2000). 11036031 10.1128/aac.44.11.3107-3111.2000 PMC101611

[b42] Pandey A. K. & Sassetti C. M. Mycobacterial persistence requires the utilization of host cholesterol. Proc Natl Acad Sci U S A 105, 4376–4380, 10.1073/pnas.0711159105 (2008). 18334639 PMC2393810

[b43] Kim M. J. *et al.* Caseation of human tuberculosis granulomas correlates with elevated host lipid metabolism. EMBO molecular medicine 2, 258–274, 10.1002/emmm.201000079 (2010). 20597103 PMC2913288

[b44] Cruz D. *et al.* Host-derived oxidized phospholipids and HDL regulate innate immunity in human leprosy. The Journal of clinical investigation 118, 2917–2928, 10.1172/JCI34189 (2008). 18636118 PMC2467381

