# Supplementary material for: Analysis of the metabolome of Anopheles gambiae mosquito after exposure to Mycobacterium ulcerans
Source: Sci Rep. 2015 Mar 18;5:9242. doi: 10.1038/srep09242 (PMC4363836; doi:10.1038/srep09242)
Supplement: Supplementary Information [file srep09242-s1.pdf]

Analysis of the metabolome of *Anopheles gambiae* mosquito after exposure to *Mycobacterium ulcerans*

J. Charles Hoxmeier[1#], Brice D. Thompson[1], Corey D. Broeckling[2], Pamela Small[3], Brian D. Foy[1], Jessica Prenni[2,4], Karen M. Dobos[1*]

Supplementary Figures and Table for SREP-14-07711

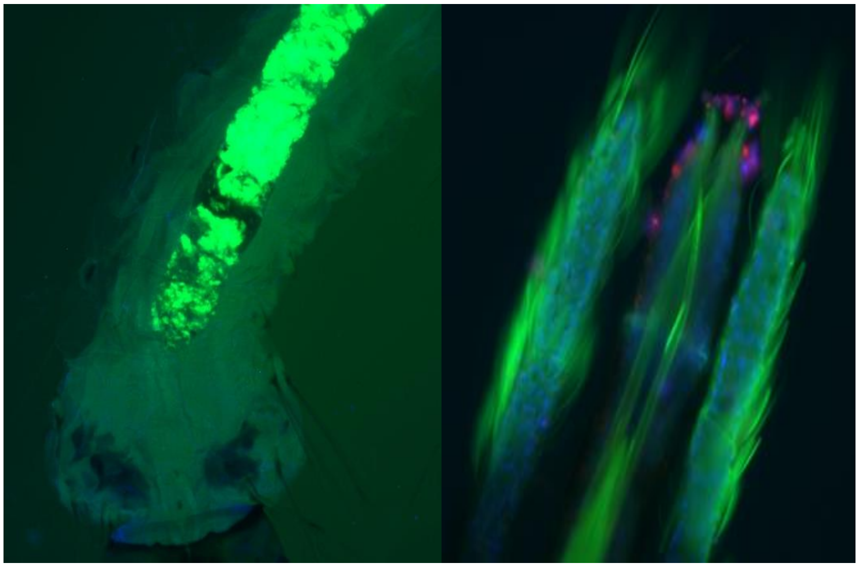

Supplemental Figure S1: Alimentary canal of larval *A. gambiae* mosquito packed with *M. ulcerans* 1615-GFP, viewed under a fluorescent microscope (10x) (Left). Immunofluorescence image of the mosquito proboscis and maxillary palps at 40x magnification. *M. ulcerans* bacilli (labelled in red) contaminated the tip of the labellum (Right).

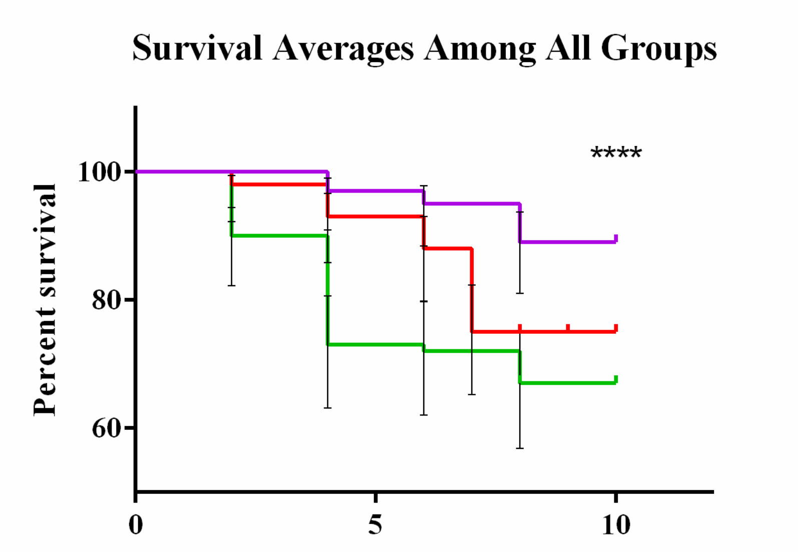

Supplemental Figure S2: Kaplan-Meier plot of Survival to adulthood for *Anopheles gambiae* mosquitoes from all treatment groups. Significant (****=*P≤0.0001)* trend for survival among all groups and significant difference (****=*P≤0.0001)* in final survival between mosquitoes exposed to live compared to dead *M. ulcerans*. Mosquitoes exposed to dead, ɣ-irradiated *M. ulcerans* (top), control mosquitoes (middle), mosquitoes exposed to live *M. ulcerans* (bottom).

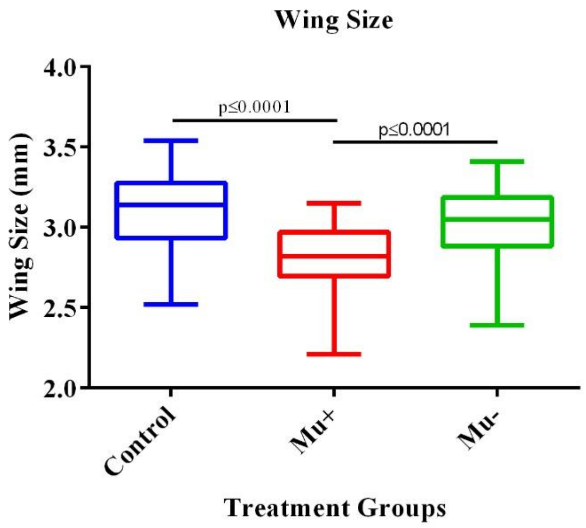

Supplemental Figure S3: Comparison of the wing size of emerged female mosquitoes as a proxy for body size. Adult mosquitoes are significantly smaller than their control counterparts as a result of exposure to live *M.*

Supplementary Table S1

ID[a]	Compound Annotation[b]	KEGG ID[c]	Retention time[d]	m/z[e]	*P*-value[f] Mu+ vs Ctrl	*P*-value[f] Mu- vs Ctrl	ID confidence[g]	Fold Change Control vs Mu+[h]	Fold Change Control vs Mu-[i]
**C186**	1-hexadecanoyl-sn-glycero-3-PC	C04317	522.7	518.325	0.036	0.012	II	1.660	0.740
**C449**	ACTH-like	C02017	508.4	468.309	0.009	0.051	II	1.356	1.410
**C794**	locustachykinin II	C16098	589.5	1065.693	0.417	0.008	II	1.121	1.712
**C104**	GPCHo (17:1(9Z)/22:5(4Z,7Z,10Z,13Z,16Z))	C05212	520.3	494.324	0.086	0.007	II	1.100	1.400
**C100**	GPCho (17:1(9Z)/22:5(4Z,7Z,10Z,16Z))	C05212	586.2	508.340	0.066	0.003	II	0.600	0.170
**C641**	Dodecylbenzenesulfonic acid	n/a	849.2	703.217	<0.001	0.001	II	0.524	0.482
**C814**	GPEtn 42:3	C04475	858.6	804.557	0.044	0.042	III	2.753	0.483
**C355**	GPEtn (20:0/20:0)	C04475	737.7	782.579	0.008	0.755	III	2.438	1.067
**C426**	Lyso-PC 24:0	C04317	575.7	570.355	0.004	0.562	III	2.215	1.114
**C686**	GPEtn 42:3	C04475	720.4	804.579	<0.001	0.691	III	2.002	1.070
**C456**	peptide fragment	n/a	556.4	546.284	0.005	0.259	III	1.874	0.807
**C465**	oxytocin-like	C00746	550.0	955.584	0.012	0.921	III	1.851	0.970
**C24**	Dodecylbenzenesulfonic acid	n/a	752.6	554.177	0.007	0.710	III	1.830	1.050
**C493**	Sphingomyelin 40:5	C00550	825.5	786.509	0.027	0.304	III	1.759	0.806
**C555**	dynorphin-like	C01574	646.2	508.341	0.006	0.049	III	1.592	1.306
**C326**	GPEtn(7:0/20:4(5E,8E,11E,14E))	C04475	513.6	472.364	0.016	0.042	III	1.550	1.415
**C52**	Lyso-PC 16:0	C05209	520.0	476.279	0.034	0.068	III	1.523	0.850
**C217**	Sphingomyelin (d19:1(4E)/26:1(17Z))	C00550	948.0	85.086	0.019	0.201	III	1.420	1.220
**C240**	Lyso-PC 18:0	C04317	618.5	524.372	0.034	0.844	III	1.400	0.978

**C71**	GPSer (15:0/25:0)	C18125	768.1	761.609	0.004	0.320	III	1.380	0.920
**C183**	Lyso-PE 18:1	C05209	586.4	959.613	0.021	0.016	III	1.350	1.570
**C575**	ACTH-like	C02017	587.9	587.940	0.255	0.037	III	1.309	1.780
**C243**	2-oleoyl glycerol	n/a	595.1	542.299	0.020	0.308	III	1.300	1.080
**C344**	ACTH-like	C02017	500.0	468.308	0.012	0.017	III	1.290	1.285
**C83**	GPEtn (5:0/24:4(5Z,8Z,11Z,14Z)	C04475	558.3	500.395	0.027	0.881	III	1.234	0.986
**C335**	GPEtn(9:0/18:3(6Z,9Z,12Z))	C04475	545.1	474.379	0.034	0.158	III	1.230	0.856
**C245**	peptide fragment	n/a	528.1	452.277	0.056	<0.001	III	1.220	1.930
**C444**	Lyso-PE 29:5	C05209	530.0	498.379	0.130	0.018	III	1.212	0.692
**C562**	Lyso-PE 20:4	C05209	574.1	361.274	0.230	0.010	III	1.202	0.395
**C560**	angiotensin-like	C15850	578.8	1031.713	0.018	0.011	III	1.197	1.376
**C107**	neurotensin-like	n/a	552.3	502.294	0.042	0.148	III	1.150	0.890
**C194**	Lyso-PE	C05209	543.5	502.294	0.05	0.05	III	1.140	0.840
**C123**	Lyso-PE 16:1	C05209	536.0	311.259	0.375	0.001	III	1.090	1.800
**C315**	isoprene	n/a	575.6	975.633	0.133	0.014	III	1.070	1.165
**C590**	Lyso-PC 20:1	C04317	520.3	266.639	0.397	0.008	III	1.060	1.360
**C790**	GPEtn 28:6	C04475	574.1	621.303	0.485	0.008	III	1.057	0.512
**C712**	adrenosterone-like	C05285	546.2	582.300	0.784	0.011	III	1.016	0.749
**C22**	Lyso-PE 16:1	C05209	517.6	474.260	0.590	0.005	III	0.980	1.610
**C120**	1-octadecanoyl-sn-glycero-3-PE	C04475	623.8	482.324	0.498	0.017	III	0.964	0.856
**C223**	Lyso-PE 21:0	C05209	264.2	520.338	0.001	0.097	III	0.951	1.122
**C205**	Oleic acid-like	C00712	749.0	283.264	0.479	0.044	III	0.943	0.851
**C203**	GPEtn(17:1(9Z)/17:1(9Z))	C04475	749.1	689.561	0.603	0.022	III	0.922	0.589

**C95**	1-octadecanoyl-sn-glycero-3-PE	C04475	716.0	357.300	0.558	0.031	III	0.921	0.642
**C234**	unknown	C11045	708.9	281.248	0.001	0.007	III	0.880	0.830
**C180**	prostaglandin-like	C00639	697.3	329.248	0.009	<0.001	III	0.870	0.670
**C408**	GPEtn (20:0/18:2)	C04475	670.1	379.283	0.352	0.001	III	0.867	0.586
**C688**	pimelic acid-like	C02656	708.8	245.227	0.001	0.023	III	0.850	0.781
**C125**	Lyso-PC 20:5	C04317	516.8	564.306	0.103	0.002	III	0.840	0.620
**C414**	4-(2-Hydroxyethyl)piperazine-1-ethanesulfonic acid	n/a	708.6	239.202	0.036	0.047	III	0.829	0.809
**C416**	GPEtn(15:0/18:2(2E,4E))	C04475	708.7	264.241	0.094	0.006	III	0.820	0.799
**C415**	3-cyclohexyl-1-propanol	n/a	708.8	83.086	0.019	0.011	III	0.818	0.809
**C93**	Androstane-like	C03772	669.9	267.212	0.004	0.056	III	0.806	0.836
**C322**	peptide fragment	n/a	534.3	641.271	0.035	0.240	III	0.796	0.907
**C38**	histamine-like	C00388	990.5	130.159	0.035	0.042	III	0.730	0.694
**C102**	Lyso-PE 16:0	C05209	528.0	245.616	0.732	0.027	III	0.730	1.600
**C151**	Lyso-PC 20:0	C04317	646.5	468.345	0.004	0.240	III	0.710	0.790
**C336**	Lyso-PE 17:1	C05209	549.6	506.323	0.040	0.004	III	0.680	0.391
**C421**	GPCho (14:0/17:1(9Z))	C05212	598.9	508.339	0.069	<0.001	III	0.654	0.213
**C589**	unknown	n/a	521.3	440.277	0.003	<0.001	IV	4.150	2.810
**C341**	unknown	n/a	484.9	199.133	0.002	<0.001	IV	3.450	2.390
**C828**	unknown	n/a	781.3	297.279	0.051	0.012	IV	3.392	0.536
**C753**	unknown	n/a	792.7	738.627	0.003	0.412	IV	2.346	1.181
**C321**	unknown	n/a	583.6	510.356	0.009	0.032	IV	2.320	1.590
**C209**	unknown	n/a	807.7	826.541	0.016	0.231	IV	2.260	0.755
**C506**	unknown	n/a	792.0	800.528	0.045	0.033	IV	2.199	0.765

**C591**	unknown	n/a	560.4	307.227	0.001	0.003	IV	2.110	1.800
**C78**	unknown	n/a	650.0	309.243	0.006	0.346	IV	1.917	0.851
**C643**	unknown	n/a	772.2	760.593	0.031	0.946	IV	1.860	0.985
**C795**	unknown	n/a	589.5	1001.670	0.119	0.008	IV	1.688	2.027
**C482**	unknown	n/a	734.1	331.263	0.005	0.982	IV	1.661	1.006
**C443**	unknown	n/a	530.8	522.379	0.002	0.042	IV	1.600	0.750
**C187**	unknown	n/a	517.5	452.277	0.298	<0.001	IV	1.570	1.050
**C338**	unknown	n/a	549.4	509.345	0.007	0.021	IV	1.560	1.490
**C703**	unknown	n/a	522.9	544.268	0.009	0.944	IV	1.560	1.011
**C750**	unknown	n/a	763.6	684.581	<0.001	0.053	IV	1.449	1.311
**C379**	unknown	n/a	774.7	712.611	0.020	0.405	IV	1.394	0.850
**C442**	unknown	n/a	536.3	544.341	0.001	0.614	IV	1.353	0.921
**C390**	unknown	n/a	916.8	804.556	0.015	0.480	IV	1.326	0.922
**C461**	unknown	n/a	540.6	498.379	0.038	0.142	IV	1.259	0.837
**C799**	unknown	C07443	528.1	253.100	0.186	0.002	IV	1.217	1.958
**C195**	unknown	n/a	548.1	466.293	0.076	0.018	IV	1.200	2.160
**C328**	unknown	n/a	520.2	516.306	0.149	0.003	IV	1.180	1.600
**C705**	unknown	n/a	520.3	517.311	0.545	0.018	IV	1.087	1.410
**C807**	unknown	n/a	520.2	1014.612	0.336	0.009	IV	1.069	1.773
**C711**	unknown	n/a	546.1	299.132	0.400	0.045	IV	1.065	0.799
**C422**	unknown	n/a	633.1	283.263	0.989	<0.001	IV	0.999	2.127
**C550**	unknown	n/a	708.6	179.143	0.471	0.028	IV	0.971	0.819
**C818**	unknown	n/a	728.8	308.265	0.651	0.011	IV	0.952	0.692

**C709**	unknown	n/a	539.7	743.395	0.283	0.026	IV	0.923	0.770
**C577**	unknown	n/a	536.3	571.290	0.225	0.033	IV	0.910	1.529
**C118**	unknown	n/a	704.2	235.169	0.367	0.017	IV	0.900	0.600
**C447**	unknown	n/a	525.6	299.628	0.086	0.011	IV	0.885	0.744
**C583**	unknown	n/a	516.7	298.626	0.189	0.010	IV	0.850	0.539
**C178**	unknown	n/a	669.9	171.122	0.024	0.047	IV	0.828	0.753
**C568**	unknown	n/a	568.3	318.131	0.118	0.014	IV	0.825	0.730
**C430**	unknown	n/a	568.6	279.232	0.017	0.035	IV	0.798	0.846
**C663**	unknown	n/a	946.6	304.299	0.017	0.356	IV	0.797	1.120
**C366**	unknown	n/a	825.0	529.881	<0.001	0.476	IV	0.783	0.958
**C704**	unknown	n/a	523.3	270.120	0.006	0.006	IV	0.760	0.851
**C227**	unknown	n/a	360.3	98.512	0.046	0.003	IV	0.743	0.770
**C548**	unknown	n/a	708.5	139.112	0.001	0.010	IV	0.727	0.663
**C177**	unknown	n/a	356.4	98.512	0.035	0.111	IV	0.710	0.790
**C593**	unknown	n/a	554.9	530.320	0.103	0.007	IV	0.670	0.279
**C578**	unknown	n/a	534.3	424.324	0.044	0.015	IV	0.488	0.275
**C528**	unknown	n/a	24.6	173.021	<0.001	0.228	IV	0.463	0.784
**C699**	unknown	n/a	517.6	398.327	0.033	0.047	IV	0.454	0.485

Supplementary Table S1 key a) Compound identifier. b) identification of compound based on match of mass spectra, m/z ratio, and/or retention time to available databases. c) compound identifier from Kyoto Encyclopedia of Genes and Genomes (www.genome.jp/kegg/) d) retention time (seconds) generated via liquid chromatography. e) mass to charge ratio of the base peak from the mass spectra. f) T-test p-value of pairwise comparison between specified treatment and control group. g) confidence level of identification based on matching of chromatographic and mass spectral characteristics for each compound to validated compound library or publicly available databases, from Sumner et al. 2007. Metabolomics. h/i) fold change in abundance of compound compared between control and treatment groups. Abbreviations: PC: phosphocholine; GPCho: glycerophosphocholine; GPEtn: glycerophosphoethanolamine; GPSer: glycerophosphoserine; ACTH: adrenocorticotropic hormone; X:N represents number of carbons to double bonds in a given compound.
